# Factors associated with disparities in out-of-pocket expenditure on dental care: results from two cross-sectional national surveys

**DOI:** 10.1186/s13584-020-00387-0

**Published:** 2020-06-17

**Authors:** Liat Orenstein, Angela Chetrit, Bernice Oberman, Michal Benderly, Ofra Kalter-Leibovici

**Affiliations:** 1 Cardiovascular Epidemiology Unit, Gertner Institute for Epidemiology and Health Policy Research, Sheba Medical Center, 52621 Ramat-Gan, Israel; 2 Biostatistics and Biomathematics Unit, Gertner Institute for Epidemiology and Health Policy Research, Sheba Medical Center, Ramat-Gan, Israel; 3 Epidemiology and Preventive Medicine Department, The Sackler Faculty of Medicine, Tel-Aviv University, Tel-Aviv, Israel

**Keywords:** Dental health, Out-of-pocket expenditure, Israel, Health insurance, Disparities

## Abstract

**Background:**

Socioeconomic differences in oral health and dental care utilization are a persistent problem in many high-income countries. We evaluated demographic, geographic and socioeconomic factors associated with disparities in households’ out-of-pocket expenditure (OOPE) on dental care, and the effect of ongoing dental health reform on these disparities.

**Methods:**

This cross-sectional analysis used data collected in two Israeli Household Expenditure Surveys conducted in 2014 and 2018. OOPE for dental care was estimated using a two-part multivariable model. A logistic regression was used to examine the likelihood of reporting any OOPE, and a log-transformed linear regression model examined the level of expenditure among those who reported any OOPE.

**Results:**

In 2018, OOPE on dental care accounted for 22% of total health expenditure for all households, whereas among those who reported dental OOPE it reached 43%. Households with children up to age 14 years reported lower OOPE, regardless of ownership of supplementary health insurance. Owning supplementary health insurance had a heterogeneous effect on the level of OOPE, with a significant increase among those with 0–8 years of education, compared to households without such insurance, but not among those of higher educational level. In 2014, Arab ethnic minority and residence in the country periphery were associated with a greater likelihood for any OOPE and higher amounts of OOPE on dental care. While the gaps between Jewish and Arab households persisted into 2018, those between peripheral and non-peripheral localities seem to have narrowed.

**Conclusions:**

The burden of dental OOPE on Israeli households remains heavy and some disparities still exist, even after the implementation of the dental health reform. Expanding the dental health reform and addressing barriers to preventive dental care, especially among Arabs and those of lower educational level, may help in reducing households’ private expenses on dental care.

## Background

Oral diseases are estimated to affect 3.9 billion people worldwide and account for 15 million disability-adjusted life-years (DALYs) globally [1]. They are also likely one of the most costly diseases to treat [1–3]. With growing recognition of oral diseases being a major public health concern, many countries now include coverage for dental services in their national health plans [4–6]. In some of those countries, the national health plan offers free or heavily-subsidized access to some dental services for the entire population, focusing on either routine treatments (e.g. Austria and Poland) [5, 7], or emergency treatments (e.g. Spain and Canadian Medicare) [5, 8]. In other countries, free access to dental services is restricted to specific vulnerable groups, such as children, the disadvantaged and the elderly (e.g. Sweden and Denmark) [5, 9]. Nevertheless, as socioeconomic inequalities in oral health and dental care utilization remain prevalent, improving access to dental services while containing healthcare costs is a continuous effort [5, 7, 10–13]. In the United States and Switzerland, where all dental care costs are privately covered, there is growing debate on economically-sustainable schemes that enable access to dental services for those most in need [14–16].

### Dental reform in Israel

For years, dental health services were not included in the list of public health services for the general population under the Israeli National Health Insurance (NHI) law. These services were subject to private funding, and could be partially or completely reimbursed either by supplementary insurance offered by one of the four not-for-profit health plans or by commercial insurance [17, 18]. Their high costs increased disparities between high-income and lower-income households, who often found it difficult to meet the payments [18–20].

In the 2008 Household Expenditure Survey, over a quarter of households had a dental care expense, with a mean monthly expenditure of $139. Among those households, OOPE on dental care constituted 3% of all consumption expenditure in the highest income quintile, compared to 7% of total expenditure in the lowest quintile. An investigation according to the type of dental treatment, concluded that household members from the two lowest income quintiles often have to forgo expensive oral surgeries and prosthodontic treatments [19]. Another analysis, based on the 2003 Israeli Social Survey, found that dental care is among the first to be curtailed by individuals facing poverty [21]. Zusman et al., who conducted a national survey among 12-year-olds, found that in localities from low socio-economic levels, the average number of damaged teeth was 2.3–3.8, as opposed to 1–1.6 among children living in higher socio-economic level localities [22]. Yet another national survey, comprising 618 mothers of children aged 5–18, found that one of the main reasons for lack of adherence to routine dental check-ups for children was the financial cost [4].

In order to reduce these disparities, a dental health reform was implemented in 2010, providing children up to 8-years old with free-of-charge preventive treatments and restorative treatments with small copayments, as part of the services provided by the health plans. The reform, which was gradually extended to include up to age 18, in the 2014 survey included children up to age 12 [23], and in the 2018 survey included children up to 15 years of age [24]. In 2019, the reform was further extended to include the elderly, by first subsidizing preventive and restorative treatments for people > 75-year-old, and later also subsidizing prosthetic treatments for people > 80-year-old [25]. Inclusion of people from age 67 is among the program’s long-term goals [26].

In Israel, there is no national surveillance system monitoring the population’s dental health status and needs in a timely manner, and only few published studies have focused on factors affecting dental care. In light of the ongoing dental health reform, we used two nationwide surveys, conducted 4 years apart, to evaluate households’ dental OOPE and its determinants, in order to gain in-depth understanding of the consequences of the dental health reform. This study provides essential updated information, may help to identify subpopulations for which OOPE on dental care are of high economic burden and assist oral and dental health policy makers to decrease inequalities related to dental health.

## Methods

### Data and sampling

We analyzed data collected in the 2014 and 2018 Household Expenditure Surveys conducted by the Israel Central Bureau of Statistics (ICBS). These were part of a series of ongoing surveys, examining Israeli households’ incomes and expenditures. The surveys’ methods have been previously described [27, 28]. In brief, the sample in both surveys was drawn in a two-phase process: first, a sample of localities was selected; then, households were sampled from the chosen localities. The sample population in 2018 was extended to include the Bedouin population in permanent localities. The surveys included a total of 8465 households in 2014 and 8792 households in 2018, representing over 2 million households in the general population. The response rates were 71 and 74% in 2014 and 2018, respectively.

A questionnaire administered by a trained interviewer included detailed questions on the household’s structure, demographic, geographic and socioeconomic characteristics of the household and its members, incomes and expenditures. Gender, marital status and ethnicity were determined according to the primary earner. Education level was chosen as the higher between the primary earner and his/her spouse, and socioeconomic status (SES) was defined according to the household’s locality. Income deciles were based on net income per standard person, including cash income (i.e. from work, property, pensions, interest or dividends and allowances), as well as non-cash income (i.e. from housing and cars owned by the household), after deducting compulsory payments (income tax, national insurance and health insurance tax). Periphery was determined by an index of the distance between household locality and the country center, constructed by the ICBS [29]. Peripheriality levels, ranging from 1 (very peripheral) to 5 (very central), were categorized into a binary variable where 1 indicated peripheral and 0 indicated non-peripheral.

In addition, each household filled a bi-weekly diary including daily expenditures for each household member and an additional questionnaire, relating to the past 3 or 12 months, which examined large or infrequent expenditures.

### Determining dental OOPE

Household members were asked to report any OOPE and reimbursements during the past 3 months, for four categories of dental care services: (1) preventive, restorative and prosthetic treatments (e.g. cleaning, fillings, root canal treatment, dental extraction, crowns and prostheses) (2); orthodontics (3); oral surgery (including dental implants); and (4) imaging in a dental imaging center. The OOPE of all household members were summed up, the amount of money reimbursed was subtracted from the total costs, and an average monthly expenditure was calculated for each of the categories and for overall dental OOPE. Data on dental OOPE was available for 8232 (98%) of the households in 2014 and for all households in 2018. All expenditures in New Israeli Shekels (NIS) were converted into US dollars as of 2014 (exchange rate 3.9 NIS = 1 USD) [30].

### Ownership of supplementary health insurance

Commercial insurance companies may offer reimbursement of dental expenses as part of an extensive health policy or a designated dental health insurance. The health plans’ supplementary health insurance programs also offer a wide range of discounts and benefits on dental services not included in the public health basket. These supplementary insurance programs are voluntary, and the health plans are obligated to insure all applicants [17, 18]. In 2014, dentistry constituted one of the leading health services reimbursed by the health plans’ supplementary health insurance. The overall net expenditure of the health plans for dentistry in that year was about $112 million, 13% of their overall net expenditures [31]. The extent of the coverage and the copayment fee vary between different policies and providers.

The majority of Israeli households purchase supplementary health insurance from their health plan (81.5%, with large differences between Arab and Jewish households [32]), which may explain the small proportion of households purchasing private dental insurance in our survey (8.6%). Moreover, in univariate analysis, ownership of private dental insurance was not significantly associated with the likelihood of any dental expense (*p *= 0.2) or with the amount of OOPE (*p* = 0.5).

Therefore, in the context of dental OOPE, we examined ownership of any kind of supplementary insurance, i.e. supplementary insurance offered by health plans or by commercial companies.

For the purpose of this analysis, ownership of supplementary health insurance was defined as a monthly expenditure of at least $3 on this item.

### Statistical analysis

We tested univariate associations between demographic, geographic and socioeconomic characteristics, and any OOPE for dental care during each survey period, and further studied these associations with the amount of OOPE in households that reported any expenditure on dental care.

Since the distribution of expenditures was highly skewed, with a substantial proportion of households reporting no OOPE for dental care during the survey’s period, we used a two-part model for multivariate analysis [33, 34]. First, a logistic regression was used to examine the likelihood of reporting any OOPE. Second, a linear regression with log-transformed outcome was used to examine associations with the amount of OOPE among those who reported expenditure on dental care. Finally, in order to present the differences in the amount of OOPE in the original metric, the percent of change, as compared to the reference category, was calculated using the following formula: (e^β^-1) × 100, where β is the regression coefficient of the relevant independent variable [35].

Multivariate analyses, conducted separately for each survey period, included household’s characteristics found to be significantly associated with the outcome variables in univariate analyses, and other characteristics known to be associated with the outcome. In order to account for the age composition and number of persons in the household, we created 18 dummy variables. Each dummy variable represents a 5-year age category (from '0-4' to '85+'), and counts the number of household members in that specific age category. Multi-co-linearity between all explanatory variables was tested using Cramér’s V statistic, and possible interactions between ownership of supplementary health insurance and other covariates were assessed by adding interaction product terms to the models.

All data analyses applied sample weights, in order to account for the complex sample design, and were performed by using SAS 9.4 (SAS institute, Cary, NC).

## Results

### Unadjusted OOPE

In 2014, OOPE on dental care constituted 23.8% of the overall household health service expenses for all households, whereas among those households who reported OOPE on dental care it accounted for almost half (48%) of their total health expenditure. Those rates were slightly lower in 2018, with dental OOPE comprising 22% of the overall health expenses for all households and 43% for those reporting any OOPE on dental care. While approximately 7% of households spent over 5% of their net cash income on dental care in 2018, large differences were observed across income deciles (11.5% of households in the second lowest income decile vs. 6.1% of households in the upper income decile).

**Fig. 1:**
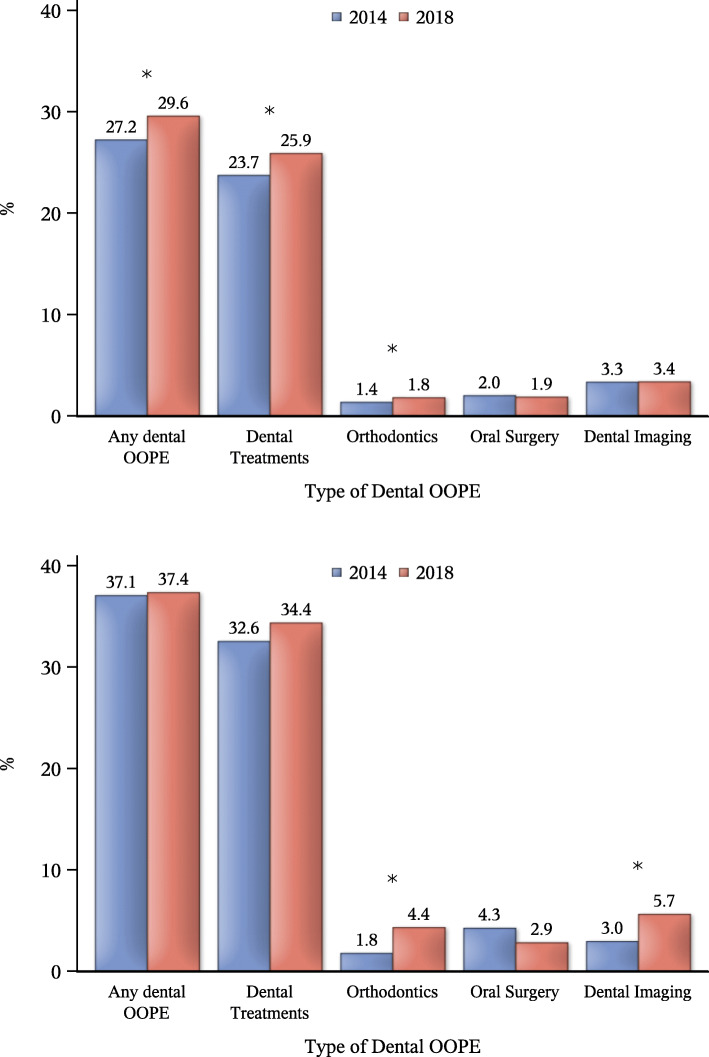
Percent of households reporting any out-of-pocket expenditure by type of dental service for **a** Jewish and **b** Arab households, separately. “Dental treatments” include preventive dentistry, such as oral hygiene cleaning treatments; *P < 0.05 according to chi-square test

Overall, the proportion of Israeli households reporting any OOPE on dental care in the 3 months preceding the survey was slightly higher in 2018 than in 2014 (30.4%, as compared to 28.0%), mainly due to an increase in the proportion of households reporting any expense on routine dental treatments (26.9% in 2018, compared to 24.5% in 2014) and on orthodontics (2.1 and 1.4%, respectively). Among Arab minority, there was no change in the percentage of households reporting any OOPE (37% in both years), while an increase was observed in the proportion of those reporting OOPE on orthodontics and on dental imaging (Fig. 1a and b).

In 2014, the share of OOPE on routine dental treatments was lower among Arab households than among Jewish households (63% of all dental-related expenditures vs. 71%, respectively), whereas the share of OOPE on oral surgery was higher (32%, as compared to 20%, respectively; *p* < 0.001). In 2018, the ethnic difference in the share of OOPE on dental treatments was somewhat smaller (68% among Arab households, as compared to 72% among Jewish households). Between the 2014 and 2018 surveys, there was a marked increase in the proportion of Arab households reporting an expense on orthodontics, making it their second largest expense (18% of all dental-related expenditures, as compared to 10% among Jewish households).

In both surveys, households with at least one child up to 14 years old were more likely to have any OOPE on dental care than those without (Table 1); however, their median monthly expenditure, as compared to households without young children, was lower ($63 compared to $71 in 2014, and $56 compared to $69 in 2018, respectively; *p* < 0.001 for both. Table 2). In addition, among households with young children, having supplementary health insurance was not associated with the amount of dental OOPE (e.g. a median of $63 regardless in 2014).

**Table 1:** Having any out-of-pocket expenditures on dental care by demographic and socioeconomic household characteristics

Characteristics	Survey year									
	2014					2018				
	Overall		Any dental OOPE		***P***	Overall		Any dental OOPE		***P***
	N	(%)	No (%)	Yes (%)		N	(%)	No (%)	Yes (%)	
	8283	(100)	5925 (72.0)	2358 (28.0)		8792	(100)	6091 (69.6)	2701 (30.4)	
Sex^†^,					< 0.001					0.017
Male	5232	(64.9)	63.5	68.5		5465	(64.8)	63.9	66.9	
Female	3051	(35.1)	36.5	31.5		3327	(35.2)	36.1	33.1	
Any household members aged 0–14,					< 0.001					< 0.001
Yes	3480	(41.0)	38.5	47.6		3547	(40.8)	37.4	48.5	
No	4803	(59.0)	61.5	52.4		5245	(59.2)	62.6	51.5	
Any household members aged 65+,					< 0.001					< 0.001
Yes	2210	(25.9)	27.7	21.2		2628	(27.9)	29.6	23.7	
No	6073	(74.1)	72.3	78.8		6164	(72.1)	70.4	76.3	
Marital Status^†^,					< 0.001					< 0.001
Married	5137	(60.0)	57.8	65.7		5359	(59.4)	56.2	66.7	
Single	1501	(20.5)	20.7	19.8		1671	(21.0)	22.0	18.4	
Divorced/Separated	1005	(11.9)	12.9	9.3		1097	(12.1)	12.9	10.4	
Widowed	640	(7.6)	8.6	5.2		660	(7.5)	8.9	4.5	
Ethnic group^†^,					< 0.001					< 0.001
Jews, of which:	6853	(83.1)	84.1	80.7		7298	(81.5)	82.5	79.2	
Born in Israel	4111	(50.7)	49.5	53.7		4532	(51.0)	50.0	53.0	
Immigrants before 1990	1334	(15.9)	16.4	14.4		1259	(13.9)	15.1	11.2	
Immigrants between 1990 and 2000	1048	(12.3)	13.3	10.0		1014	(11.5)	11.7	11.0	
Immigrants after 2000	360	(4.2)	4.9	2.6		493	(5.2)	5.6	4.1	
Arabs	1103	(13.0)	11.4	17.2		1145	(14.5)	13.1	17.9	
Others	327	(3.9)	4.5	2.1		349	(4.0)	4.4	2.9	
Religiosity,					0.1					0.067
Secular	3826	(47.2)	47.8	45.7		4055	(46.7)	47.1	45.8	
Traditional	2194	(27.5)	27.0	28.9		2370	(27.8)	28.2	27.0	
Religious	1106	(12.8)	12.7	13.0		1185	(13.2)	13.0	13.7	
Very religious/ultra-Orthodox	633	(6.1)	5	6.8		786	(7.2)	6.6	8.6	
Mixed lifestyle	524	(6.3)	6.6	5.6		396	(5.0)	5.1	4.9	
Periphery index,					0.008					0.3
Peripheral	1226	(13.1)	12.4	15.0		1484	(15.8)	15.5	16.5	
Non-peripheral	7057	(86.9)	87.6	85.1		7308	(84.2)	84.5	83.5	
Income deciles,					< 0.001					< 0.001
1–2 (Low)	1767	(19.2)	19.6	18.3		1811	(20.0)	21.0	17.7	
3–4	1683	(19.8)	20.5	18.0		1781	(20.0)	20.6	18.6	
5–6	1576	(20.2)	20.8	18.9		1718	(20.0)	20.2	19.6	
7–8	1594	(20.4)	20.7	19.6		1716	(20.0)	20.1	19.7	
9–10 (High)	1663	(20.4)	18.5	25.2		1766	(20.0)	18.1	24.4	
Years of education^‡^,					< 0.001					< 0.001
0	88	(1.1)	1.1	1.1		113	(1.2)	1.3	0.8	
1–8	432	(4.7)	5.5	2.7		399	(4.7)	5.6	2.8	
9–12	2543	(32.0)	32.3	31.1		2597	(30.7)	32.1	27.5	
13–15	1855	(22.5)	23.7	19.3		1854	(21.2)	21.8	19.8	
16+	3365	(39.7)	37.4	45.8		3829	(42.2)	39.2	49.0	
SES index^#^,					< 0.001					< 0.001
1 (Low)	590	(6.2)	5.1	9.0		654	(7.1)	6.2	9.2	
2	1859	(22.6)	21.5	25.2		1882	(21.5)	21.3	21.9	
3	3339	(39.9)	42.6	33.1		3624	(41.7)	43.5	37.6	
4	2391	(30.1)	29.8	30.8		2505	(28.5)	27.8	29.9	
5 (High)	104	(1.2)	1.0	1.7		127	(1.2)	1.2	1.4	
Ownership of an apartment,					< 0.001					< 0.001
Yes	5717	(67.9)	65.7	73.7		6030	(66.5)	64.1	71.9	
No	2566	(32.1)	34.3	26.3		2762	(33.5)	35.9	28.1	
Ownership of a car,					< 0.001					< 0.001
No	2606	(30.8)	33.7	23.2		2489	(27.9)	31.8	19.0	
One car	3760	(44.8)	44.2	46.6		3990	(45.7)	45.1	46.8	
Two cars or more	1917	(24.4)	22.1	30.3		2313	(26.5)	23.1	34.1	
Ownership of supplementary health insurance,					1.0					< 0.001
Yes	6978	(84.1)	84.2	84.1		7394	(82.3)	80.9	85.3	
No	1305	(15.9)	15.8	15.9		1398	(17.7)	19.1	14.7	

*OOPE* out-of-pocket expenditure, *SES* socioeconomic status

Notes: Dental care includes dental treatments, orthodontics, oral surgery and dental imaging; Percentages are weighted in order to represent the entire Israeli population; Income deciles are based on net income per standard person deciles, including cash income (i.e. from work, property, pensions, interest or dividends and allowances), as well as non-cash income (i.e. from housing and cars owned by the household), after deducting compulsory payments (income tax, national insurance and health insurance tax); Peripherality index incorporates the potential accessibility of the locality as well as its proximity to the boundary of the Tel Aviv District; Supplementary insurance includes supplemental insurance of the health plans, private commercial health insurance, private dental insurance, long-term care insurance and other private health insurance

^†^ Defined according to the primary earner

^‡^ Defined according to the highest between the primary earner and his/her spouse

^#^ Defined according to the locality in which the household resides

**Table 2:** Monthly out-of-pocket expenditures on dental care by demographic and socioeconomic household characteristics

Characteristics	Survey year					
	2014			2018		
	Monthly dental OOPE in USD		***P***	Monthly dental OOPE in USD		***P***
	N	Median (Q1;Q3)		N	Median (Q1;Q3)	
	2358	68 (21;190)		2701	64 (21;196)	
Sex^†^,			0.5			0.4
Male	1581	68 (21;179)		1728	67 (21;198)	
Female	777	68 (19;216)		973	60 (21;181)	
Any household members aged 0–14,			< 0.001			< 0.001
Yes	1144	63 (20;171)		1304	56 (19;170)	
No	1214	71 (21;229)		1397	69 (21;213)	
Any household members aged 65+,			< 0.001			< 0.001
Yes	532	85 (24;256)		677	85 (26;273)	
No	1826	60 (18;172)		2024	59 (19;170)	
Marital Status^†^,			0.6			0.4
Married	1590	74 (22;192)		1834	66 (21;204)	
Single	417	53 (17;181)		456	51 (17;163)	
Divorced/Separated	224	60 (17;221)		284	61 (17;179)	
Widowed	127	48 (17;171)		124	85 (26;230)	
Ethnic group^†^,			< 0.001			< 0.001
Jews, of which	1907	63 (18;179)		2192	59 (18;175)	
Born in Israel	1245	58 (17;172)		1459	52 (17;170)	
Immigrants before 1990	353	76 (23;214)		329	86 (23;256)	
Immigrants between 1990 and 2000	241	59 (17;177)		292	44 (16;150)	
Immigrants after 2000	68	60 (19;218)		112	81 (23;188)	
Arabs	396	102 (26;256)		431	85 (34;256)	
Others	55	68 (21;102)		78	72 (28;212)	
Religiosity,			< 0.001			< 0.001
Secular	1061	64 (20;181)		1213	52 (17;172)	
Traditional	655	85 (25;214)		713	85 (25;255)	
Religious	316	51 (17;171)		368	59 (20;181)	
Very religious/ultra-Orthodox	198	51 (17;128)		290	51 (20;156)	
Mixed lifestyle	128	60 (20;248)		117	68 (29;183)	
Periphery index,			< 0.001			0.003
Peripheral	387	120 (34;257)		480	69 (26;213)	
Non-peripheral	1971	60 (18;172)		2221	60 (19;192)	
Income deciles,			0.034			0.15
1–2 (Low)	486	55 (17;171)		508	60 (21;170)	
3–4	435	65 (20;171)		510	60 (21;170)	
5–6	426	60 (17;224)		517	69 (19;183)	
7–8	442	77 (21;184)		518	64 (21;213)	
9–10 (High)	569	68 (22;229)		648	67 (21;215)	
Years of education^‡^,			< 0.001			< 0.001
0	21	43 (14;254)		24	112 (25;239)	
1–8	77	93 (30;179)		72	66 (33;256)	
9–12	696	85 (22;256)		718	85 (25;255)	
13–15	465	67 (21;172)		539	69 (21;201)	
16+	1099	55 (18;170)		1348	51 (17;166)	
SES index^#^,			0.005			< 0.001
1 (Low)	230	103 (30;256)		261	99 (29;255)	
2	583	60 (19;209)		586	69 (23;175)	
3	796	77 (20;191)		997	68 (20;213)	
4	705	54 (19;172)		814	44 (17;160)	
5 (High)	44	52 (14;127)		43	78 (22;306)	
Ownership of an apartment,			< 0.001			< 0.001
Yes	1757	77 (22;222)		1979	69 (21;207)	
No	601	43 (17;145)		722	51 (17;172)	
Ownership of a car,			0.055			0.3
No	578	51 (17;171)		548	69 (21;180)	
One car	1096	76 (21;217)		1252	64 (21;207)	
Two cars or more	684	67 (21;178)		901	59 (21;181)	
Ownership of supplementary health insurance,			0.2			0.09
Yes	2010	71 (21;199)		2349	64 (21;196)	
No	348	51 (18;171)		352	68 (25;184)	

*OOPE* out-of-pocket expenditure, *Q1* first quartile, *Q3* third quartile, *SES* socioeconomic status

Notes: Dental care includes dental treatments, orthodontics, oral surgery and dental imaging; ‘Monthly dental OOPE in USD’ is analyzed only for households who reported any; Income deciles are based on net income per standard person deciles, including cash income (i.e. from work, property, pensions, interest or dividends and allowances), as well as non-cash income (i.e. from housing and cars owned by the household), after deducting compulsory payments (income tax, national insurance and health insurance tax); Peripherality index incorporates the potential accessibility of the locality as well as its proximity to the boundary of the Tel Aviv District; Supplementary insurance includes supplemental insurance of the health plans, private commercial health insurance, private dental insurance, long-term care insurance and other private health insurance

^†^ Defined according to the primary earner

^‡^ Defined according to the highest of the primary earner and his/her spouse

^#^ Defined according to the locality in which the household resides

Households with at least one member aged 65 years or older were less likely to report dental-related OOPE; however, those that did, spent a significantly higher median amount ($85, compared to $59–$60 among households without elderly members, in both surveys; *p* < 0.001).

The proportion of Arab households reporting any OOPE on dental care was about 10% higher in 2014 and 8% higher in 2018 than among Jewish households (*p* < 0.001 for both). Among households that reported any amount of dental OOPE, Arab households had a substantially higher monthly OOPE. In 2014 they reported a median of $102, compared to $63 among Jewish households, and in 2018 they spent a median of $85 compared to $59 among Jewish households (*p* < 0.001 for both).

In 2014, households residing in peripheral localities were more likely to have OOPE on dental care, and spent a substantially higher monthly amount ($120, compared to $60 among other households; *p* < 0.001). In contrast, in 2018 there was no difference between peripheral and non-peripheral localities in reporting any dental OOPE (*p* = 0.3). Further analysis showed that, among households residing in the periphery, the only significant difference between the two surveys’ periods was a decrease in the proportion of those reporting OOPE on oral surgery (from 4.0% in 2014 to 2.1% in 2018; *p* = 0.01). In addition, while the median expenditure of households from non-peripheral localities did not change between the two surveys’ periods, the median expenditure was reduced in 2018 almost by half ($69) among those leaving in peripheral localities.

Sociodemographic characteristics were also associated with OOPE on dental care in both surveys. Households in the two upper income deciles, and households who own their home were more likely to report any OOPE and spent more compared to other households. Households with the highest education level (i.e. primary earner or his/her spouse had at least 16 years of education) were more likely to report any dental OOPE; however there was an inverse association between educational level and the amount of OOPE. Finally, a U shaped relationship was observed between the locality’s socioeconomic index and the probability for reporting any OOPE, with the highest rates among the lowest and the highest SES groups. In addition, an inverse association was found between SES level and the reported amount of OOPE.

While in 2014 ownership of supplementary health insurance was not associated with any OOPE or with the amount of OOPE on dental care, in 2018, the proportion of households reporting a dental expense was higher among those who owned supplementary health insurance (*p* < 0.001).

### Adjusted OOPE

In the multivariable logistic regression models of both surveys’ periods, educational level, income level and ethnicity were significantly and independently associated with any OOPE on dental care (Table 3). Households with the highest education level and those in the two highest income deciles were more likely to report any OOPE, compared to households in the lowest education level (Odds Ratio (OR) = 1.81, 95%Confidence Interval (CI): 1.32–2.49, in 2018) and those in the two lowest income deciles (OR = 1.74, 95%CI: 1.37–2.22, in 2018), respectively.

**Table 3:** Association of demographic and socioeconomic characteristics with the likelihood for reporting any out-of-pocket expenditure for dental care: multivariate analysis

Independent variable	Survey year					
	2014			2018		
	OR	95% CI	P	OR	95% CI	***P***
Sex			0.6			0.022
Female	1.00	Ref. cat.		1.00	Ref. cat.	
Male	0.97	(0.85–1.11)		0.86	(0.76–0.98)	
Marital status			0.3			0.6
Single	1.00	Ref. cat.		1.00	Ref. cat.	
Married	0.86	(0.72–1.02)		1.05	(0.89–1.24)	
Divorced/Separated	0.83	(0.66–1.05)		1.01	(0.75–1.34)	
Widowed	0.89	(0.66–1.19)		1.01	(0.75–1.34)	
Religiosity level			0.5			0.2
Secular	1.00	Ref. cat.		1.00	Ref. cat.	
Traditional	1.04	(0.90–1.20)		0.92	(0.80–1.06)	
Religious	0.94	(0.77–1.14)		0.98	(0.81–1.17)	
Very religious/ultra-Orthodox	1.10	(0.85–1.43)		1.22	(0.96–1.54)	
Mixed lifestyle	0.85	(0.65–1.10)		0.88	(0.66–1.17)	
Years of education			< 0.001			< 0.001
0–8	1.00	Ref. cat.		1.00	Ref. cat.	
9–12	1.27	(0.93–1.72)		1.29	(0.95–1.77)	
13–15	1.27	(0.92–1.75)		1.50	(1.09–2.08)	
16+	1.72	(1.25–2.37)		1.81	(1.32–2.49)	
Income deciles			< 0.001			< 0.001
1–2 (Low)	1.00	Ref. cat.		1.00	Ref. cat.	
3–4	1.15	(0.93–1.42)		1.19	(0.98–1.45)	
5–6	1.26	(1.00–1.59)		1.30	(1.05–1.61)	
7–8	1.28	(1.00–1.63)		1.25	(0.99–1.56)	
9–10 (High)	1.76	(1.36–2.28)		1.74	(1.37–2.22)	
Peripherality index			0.040			0.7
Non-peripheral	1.00	Ref. cat.		1.00	Ref. cat.	
Peripheral	1.19	(1.01–1.41)		0.97	(0.83–1.16)	
Ownership of an apartment			0.8			0.9
No	1.00	Ref. cat.		1.00	Ref. cat.	
Yes	0.98	(0.85–1.14)		1.01	(0.87–1.16)	
Ownership of a car			0.2			0.008
No	1.00	Ref. cat.		1.00	Ref. cat.	
One car	1.17	(0.99–1.38)		1.28	(1.08–1.50)	
Two cars or more	1.19	(0.96–1.47)		1.36	(1.11–1.67)	
Ethnicity	Interaction results are presented in Table 4		0.003			< 0.001
Jewish				1.00	Ref. cat.	
Arab				1.77	(1.43–2.18)	
Other				0.87	(0.64–1.19)	
Ownership of supplementary health insurance						0.022
No				1.00	Ref. cat.	
Yes				1.25	(1.03–1.51)	

*OR* odds-ratio, *CI* confidence interval, *Ref. cat.* reference category

Notes: Weighted models, adjusted for the age composition of the household; c-Statistic = 0.641 (95%CI: 0.628–0.654) for 2014 survey and 0.646 (95%CI: 0.634–0.658) for 2018 survey; Income deciles are based on net income per standard person deciles, including cash income (i.e. from work, property, pensions, interest or dividends and allowances), as well as non-cash income (i.e. from housing and cars owned by the household), after deducting compulsory payments (income tax, national insurance and health insurance tax); Peripherality index incorporates the potential accessibility of the locality as well as its proximity to the boundary of the Tel Aviv District; Supplementary insurance includes supplemental insurance of the health plans, private commercial health insurance, private dental insurance, long-term care insurance and other private health insurance

A significant interaction was found in 2014 between ethnicity and ownership of supplementary health insurance (*p* = 0.003; Table 4). Arab households that did not own supplementary health insurance were 2.4 times more likely to report OOPE on dental care (95%CI: 1.62–3.58), compared to Jewish households. However, ownership of supplementary insurance attenuated this ethnic difference in the likelihood to report any OOPE (OR = 1.21 for Arab as compared to Jewish households, 95%CI: 0.88–1.67). While ownership of such insurance did not affect the likelihood of spending any out-of-pocket money on dental care among Jews, it reduced this likelihood for Arabs by 44% (95%CI: 0.39–0.80). In 2018 survey, no such interaction was observed. Arab households were more likely to report any dental OOPE (OR = 1.77, 95%CI: 1.43–2.18); and ownership of supplementary health insurance was associated with a 25% increase in the likelihood for reporting OOPE, for all households (95%CI: 1.03–1.51).

**Table 4:** Interaction between ownership of supplementary health insurance and ethnicity, and their effect on the possibility of having any out-of-pocket expenditure for dental care (2014 survey)

	Ownership of a supplementary health insurance		OR (95% CI) for with supplementary insurance ***(***vs ***without)*** stratified by ethnicity
Ethnicity	No (***N*** = 1305)	Yes (***N*** = 6978)	
	OR (95% CI)	OR (95% CI)	
**Jewish (N = 6853)**	1.0 Reference category	1.10 (0.87–1.41)	
**Arab (N = 1103)**	2.40 (1.62–3.58)	1.34 (0.94–1.89)	**0.56 (0.39–0.80)**
**OR (95% CI) for Arab*(*vs*Jewish)*stratified by ownership of supplementary insurance**		**1.21 (0.88–1.67)**	

*OR* odds-ratio, *CI* confidence interval

Notes: Weighted model, adjusted for sex, marital status, religiosity level, years of education, income deciles, peripherality index, ownership of an apartment, ownership of a car, and the age composition of the household; Supplementary insurance includes supplemental insurance of the health plans, private commercial health insurance, private dental insurance, long-term care insurance and other private health insurance; Values are presented after Bonferroni correction for multiple-comparisons

Among households that reported any OOPE, Arab ethnicity was significantly associated with higher level of OOPE for dental services in both surveys (Table 5). Compared to Jewish households, Arab households had a 52% higher OOPE in 2014, and 82% in 2018 (*p* = 0.017 and *p* < 0.001, respectively).

**Table 5:** Association of demographic and socioeconomic characteristic with the level of out-of-pocket expenditure for dental care: multivariate analysis

Independent variable	Survey year					
	2014			2018		
	OOPE for dental care		***P***	OOPE for dental care		***P***
	Log coefficient (SE)	% change (95% CI)		Log coefficient (SE)	% change (95% CI)	
Sex			0.1			0.7
Female	0	Ref. cat		0	Ref. cat	
Male	−0.134 (0.084)	−12.50 (−25.80–3.18)		−0.029 (0.073)	− 2.86 (− 15.86–12.15)	
Marital Status			0.2			0.2
Single	0	Ref. cat.		0	Ref. cat	
Married	0.235 (0.113)	26.45 (1.40–57.67)		0.222 (0.102)	24.86 (2.16–52.61)	
Divorced/Separated	0.162 (0.166)	17.54 (−15.17–62.86)		0.180 (0.133)	19.74 (−7.67–55.29)	
Widowed	0.045 (0.204)	4.62 (−29.83–55.98)		0.215 (0.185)	24.05 (−13.64–78.18)	
Ethnicity			0.017			< 0.001
Jewish	0	Ref. cat.		0	Ref. cat	
Arab	0.418 (0.151)	51.93 (12.93–104.39)		0.598 (0.121)	81.85 (43.49–130.45)	
Other	−0.106 (0.192)	−10.09 (−38.29–30.99)		0.199 (0.177)	22.03 (−13.76–72.67)	
Religiosity level			0.5			0.064
Secular	0	Ref. cat.		0	Ref. cat	
Traditional	0.040 (0.093)	4.11 (−13.18–24.83)		0.211 (0.085)	23.45 (4.47–45.88)	
Religious	−0.136 (0.126)	−12.75 (−31.90–11.78)		0.019 (0.106)	1.94 (−17.15–25.43)	
Very religious/Orthodox	−0.065 (0.164)	−6.30 (−32.06–29.23)		0.222 (0.131)	24.88 (−3.41–61.45)	
Mixed lifestyle	0.169 (0.164)	18.40 (−14.08–63.16)		0.166 (0.173)	18.11 (−15.91–65.88)	
Income deciles			0.3			0.004
1–2 (Low)	0	Ref. cat.		0	Ref. cat	
3–4	0.050 (0.126)	5.09 (−17.90–34.51)		0.200 (0.112)	22.15 (−1.97–52.19)	
5–6	0.081 (0.149)	8.47 (−18.97–45.21)		0.298 (0.124)	34.72 (5.72–71.68)	
7–8	0.238 (0.152)	26.87 (−5.78–70.83)		0.488 (0.133)	62.89 (25.62–111.21)	
9–10 (High)	0.231 (0.156)	26.00 (−7.23–71.14)		0.487 (0.143)	62.78 (23.10–115.26)	
Peripherality index			< 0.001			0.09
Non-peripheral	0	Ref. cat.		0	Ref. cat	
Peripheral	0.408 (0.111)	50.40 (20.90–87.10)		0.150 (0.088)	16.13 (−2.36–38.14)	
Ownership of an apartment			0.6			0.2
No	0	Ref. cat.		0	Ref. cat	
Yes	0.054 (0.098)	5.59 (−12.80–27.85)		−0.121 (0.088)	−11.37 (− 25.37–5.27)	
Ownership of a car			0.4			0.2
No	0	Ref. cat.		0	Ref. cat	
One car	0.074 (0.106)	7.65 (−12.51–32.45)		−0.147 (0.099)	−13.65 (−28.91–4.88)	
Two cars or more	−0.046 (0.129)	−4.47 (− 25.84–23.05)		− 0.198 (0.121)	−17.94 (−35.26–4.01)	
Years of education			0.4	Interaction results are presented in Table 6		0.009
0–8	0	Ref. cat.				
9–12	0.138 (0.205)	14.84 (−23.23–71.81)				
13–15	−0.006 (0.231)	−0.56 (−36.37–56.47)				
16+	−0.024 (0.218)	−2.34 (−36.37–49.88)				
Ownership of supplementary health insurance			0.043			
No	0	Ref. cat.				
Yes	0.253 (0.125)	28.80 (0.76–64.63)				

*OOPE* out-of-pocket expenditure, *SE* standard error, *CI* confidence interval, *Ref. cat.* reference category

Notes: Weighted models, adjusted for the age composition of the household; Adjusted R-square = 0.068 for 2014 and 0.063 for 2018; % change in USD, as calculated by the formula: (e^β^-1) × 100; Income deciles are based on net income per standard person deciles, including cash income (i.e. from work, property, pensions, interest or dividends and allowances), as well as non-cash income (i.e. from housing and cars owned by the household), after deducting compulsory payments (income tax, national insurance and health insurance tax); Peripherality index incorporates the potential accessibility of the locality as well as its proximity to the boundary of the Tel Aviv District; Supplementary insurance includes supplemental insurance of the health plans, private commercial health insurance, private dental insurance, long-term care insurance and other private health insurance

In 2014, ownership of supplementary health insurance was associated with an approximate 29% increase in the level of OOPE on dental care (*p* = 0.043). However, in 2018 an interaction was found between ownership of supplementary health insurance and educational level (Table 6). Ownership of such insurance increased the level of OOPE among those with 0–8 years of education, but did not affect the level of OOPE among households with a higher educational level.

**Table 6:** Interaction between education and ownership of supplemental health insurance, and their effect on the level of out-of-pocket expenditure for dental care (2018 survey)

	Ownership of a supplementary health insurance among those with an expense		Log coefficient (SE) for with supplementary insurance ***(***vs ***without)*** stratified by education years
Years of education	No (***N*** = 352)	Yes (***N*** = 2349)	
	Log coefficient (SE)	Log coefficient (SE)	
**0–8 (N = 96)**	1.0 Reference category	1.11 (0.32)*	
**9–12 (N = 718)**	0.61 (0.28)		**0.24 (0.16)**
**13–15 (N = 539)**	0.94 (0.32)*		**−0.18 (0.23)**
**16+ (N = 1348)**	0.58 (0.35)		**0.06 (0.23)**
**Log coefficient (SE) for 9–12*(*vs*0–8)*stratified by ownership of supplementary insurance**		**−0.27 (0.24)**	
**Log coefficient (SE) for 13–15*(*vs*0–8)*stratified by ownership of supplementary insurance**		**−0.36 (0.24)**	
**Log coefficient (SE) for 16+*(*vs*0–8)*stratified by ownership of supplementary insurance**		**−0.48 (0.24)**	

*SE* standard error

*P < 0.05 after Bonferroni correction for multiple-comparisons

Notes: Weighted model, adjusted for sex, marital status, religiosity level, ethnicity, income deciles, peripherality index, ownership of an apartment, ownership of a car, and the age composition of the household; Supplementary insurance includes supplemental insurance of the health plans, private commercial health insurance, private dental insurance, long-term care insurance and other private health insurance

**Fig. 2:**
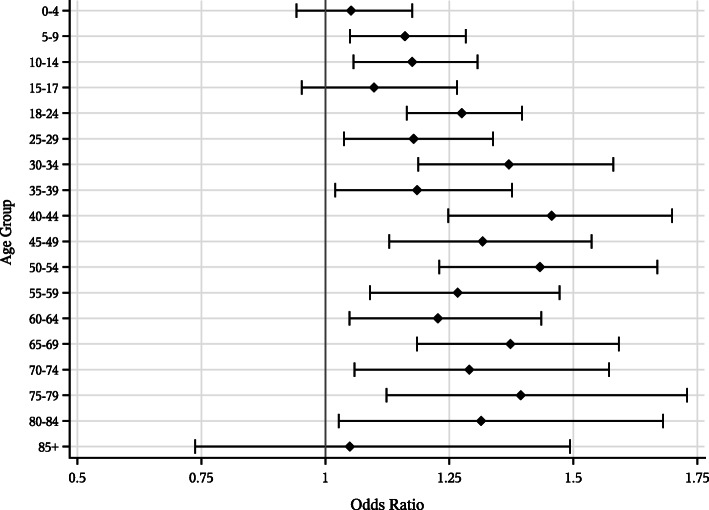
The effect of household’s age composition on the likelihood of having any dental out-of-pocket expenditure. Odds Ratio: Adjusted odds ratio (95% confidence interval) from the multivariable logistic regression model for 2018. In each age group, the risk is associated with an increase of one household member in those specific ages

After accounting for demographic and socioeconomic factors, the age composition of the household was still significantly associated with the likelihood for any OOPE on dental care, in both surveys. The highest OR (1.45) in 2018 was observed for those aged 40–44 (i.e. each additional household member in this age group increased the risk by 1.45; Fig. 2**)**. The age composition of the household was also associated with the level of dental OOPE, with a steady increase in the level of OOPE between ages 50 to 84 years.

OOPE for dental care in peripheral localities was 50% higher compared to other localities in 2014 (*p* < 0.001). However in 2018, the difference between peripheral and non-peripheral localities was no longer statistically significant.

Finally, while in 2014 the household’s income level was not significantly associated with the level of dental OOPE, in 2018 the gap in dental expenses between the upper and lower income deciles increased. As compared to households in the two lowest income deciles, the level of expenditure was 63% higher for those in income deciles 7 and above (*p* = 0.004).

## Discussion

The present study found that the economic burden of OOPE on dental care remains high even with partial coverage of dental expenses. While OOPE for dental services constitutes about 22% of all households’ total health expenses, it accounts for over two-fifths of total health expenses among those who reported any OOPE.

We found that households with at least one member aged 0–14 years were more likely to have any dental OOPE; however the level of their OOPE, compared to households without young children, was lower. Moreover, no difference was found in the amount of OOPE by ownership of any supplementary health insurance, which implies reliance on publicly-funded services. This finding illustrates the contribution of dental health reform in reducing the burden of dental care expenditure among households with young children. This finding is in accordance with a previous study [4], reporting an increase in children’s dental services utilization following the same dental health reform, especially among the less privileged population segments. Other countries have recognized the importance of dental care for the pediatric population. Some have included free dental services for children in their national health plan, as described above. In Australia, a new publicly funded dental program for children aged 2–17 years was introduced in 2014 under the Child Dental Benefits Schedule (CDBS) [11]. In the US, there are oral health programs for disadvantaged children, such as Medicaid and the Children’s Health Insurance Program (CHIP) [14], and since 2014 children’s dental coverage is included in the Affordable Care Act (Obama Care) [4]. Recently, a Canadian study found that children with poor oral health were more likely to visit dentists in jurisdictions with public dental care programs for children [36].

An opposite trend was observed among households with at least one person aged 65 years or older: fewer such households reported having any dental OOPE; however among those who did, the level of OOPE was significantly higher. The higher dental OOPE among older people may reflect age-related increase in the prevalence of dental disease, due to natural physiological processes, periodontal disease associated with chronic morbidity, etc [37]. It is also possible that older people are less likely to invest in preventive and restorative dental treatments, and visit their dentist only if there is a more serious condition, often needing more complex and expensive care. A partial explanation for the high dental expenses among older adults may stem from the prevalence of edentate patients in this age group. As previously mentioned, during the surveys’ period (2014–2018) older people were still not included in the dental health reform. Even with a coverage of a supplementary health insurance, the copayments for the more complex prosthetic treatments were still high. Sgan-Cohen et al. [38] showed that Israel, compared to 14 other European countries, has the second-worst income-related inequality for use of dental treatments among ages 50 and above, and the worst income-related level of inequality in chewing ability, an index of dental health. During the years 2008–2011 the “Smile Again” project, a collaborative project of the Health, Social Services and Senior Citizens Ministries, was implemented [39]. It consisted of providing dental treatment to needy elderly, of which about 2000 were community-dwelling and nearly 500 were living in long-term care institutions. Analysis of this project data emphasized the high rate of unmet dental needs in this elderly population, with prosthetic treatments provided at the highest rates (83% of the treatments among people living in the community, and 92% of treatments among those living in long-term care institutions). Recently, Shahrabani [40] found that 53% of people 50–75 years old reported having a routine dental checkup once every 2 years or less often. The main reason for avoiding routine dental checkups was the financial cost. Although the recent extension of the dental reform to include people aged 75 and older takes a step in the right direction, we recommend including also people 50–74 years old.

The level of expenditure on dental care was significantly higher among Arab households. A lower accessibility and availability of service providers under public funding and a lower rate of purchasing supplementary insurance among Arabs, could eventually lead to the neglect of preventive care and the need for more complex and expensive dental treatments later on [20, 32, 41–49]. This is further supported by our finding from the 2014 survey that the share of OOPE on routine dental treatments was lower among Arab households than among Jewish households, whereas the share of OOPE on oral surgery was higher. Other barriers may be also cultur-related [44]. The even larger gap in the level of dental OOPE between Jewish and Arab households seen in 2018 may be due to the increase in the proportion of households reporting an expense on orthodontics. As children aged 13–15 years were added to the dental health reform between the two surveys’ periods, more children of those ages now saw a dentist during the free routine checkups, and more were referred to orthodontic treatments. It could also be that with the subsidized preventive and restorative treatments for children, more households could now afford orthodontic treatments. The increase in the proportion of households with an expense on orthodontics, which is relatively expensive and not covered by the reform, was more substantial in Arab households. It should be noted that a report bias might have contributed to the observed disparities between Arabs and Jews, as cultural differences may lead to differential reporting of expenditures [50].

Ownership of supplementary health insurance attenuated education-related disparities in the expenditure on dental care in the 2018 survey. While it had no effect on households with a higher educational level, it increased the amount of OOPE for those with 0–8 years of education. It is possible that without such insurance low-education households could not afford complex and costly treatments and were forced to forgo them. Another possible explanation may stem from the extension of the dental health reform. We showed earlier that a lower educational level was associated with a lower likelihood for having any dental OOPE, probably due to lower awareness of the importance of dental health and lower ability to pay for the treatments [19, 45]. It is possible, as explained above, that due to the further extension of the reform in 2018, more children were now referred to follow-up treatments and orthodontics, while those with a supplemental insurance could more often afford those additional treatments. It is also possible, in light of the cross-sectional design, that those who anticipate need for expensive treatments purchase supplementary insurance in the first place [19, 51]. In the Israeli population, educational level and ethnicity are highly correlated, as there is a lower educational level among the Arab minority [52]. Similarly, in the 2014 survey, ownership of a supplementary health insurance attenuated ethnic-related disparities in the likelihood of any dental-related OOPE.

Households residing in the periphery had both a higher likelihood and higher amount of reported OOPE on dental care in 2014. It is possible that lower availability of publicly-funded services in peripheral areas and a lower proportion of households purchasing supplementary insurance [32, 45, 46], led to lower utilization rates of preventive dental care, thereby contributing to an increase in the share of the more complex and expensive treatments. The 2018 data showed that the differences between households residing in peripheral and non-peripheral localities were attenuated. This may be a result of the expansion of the dental health reform, as more publicly-funded dental services were now available in the periphery. Another factor contributing to the gap between the two surveys may be the time lag between the legislation and the implementation of the dental health reform resulting in a behavioral response of the public. Moreover, a change in the types of treatments was observed between the two surveys according to residential area. The increase in the proportion of households reporting an expense on orthodontics, which was seen in the general population, was not seen in the periphery. In addition, a decrease in the proportion of those reporting OOPE on oral surgery was observed in the periphery only. The lower tendency to purchase expensive dental treatments in the periphery, even at the cost of a serious and immediate impact on one’s well-being, as is often the case in oral surgery, may be due to financial difficulties that deepened during those 4 years.

It should be emphasized that our study examined private expenditure and disparities in the economic burden of dental care, and did not directly measure dental health status or utilization of dental services. Reporting no dental OOPE, for example, may be due to good oral health or full coverage by supplemental insurance on the one hand; or due to lack of financial resources and/or dental health awareness on the other. The availability of two time-points for comparison in our study helped in the interpretation of the results, however, the direct impact of the dental health reform on disparities in oral health should be further examined in future studies.

This study has some limitations. First, the study was based on a cross-sectional survey, where responders were asked about any dental related OOPE in the last 3 months. This design may be affected by recall bias, reporting bias and reverse-causality [50], as described above. Second, due to the relatively small number of households reporting any dental OOPE, we were unable to explore expenditure differences related to type of dental service. Third, the survey did not capture group supplementary insurance plans provided by employers, which are not privately funded. Another limitation is a difference in the composition of Arab households that were sampled between the two surveys, where the Bedouin population in permanent localities were sampled only in 2018 survey. However, a sensitivity analysis, excluding the Be’er Sheva Subdistrict, where the majority of Bedouins reside, showed similar associations (data not shown). Finally, the predictive accuracy of the logistic models (c-Statistics = 0.641–0.646) and the percentage of the response variable variation that is explained by the linear models (about 6–7%) are relatively low, suggesting that a substantial portion of the differences between households in the likelihood of any OOPE and in the level of OOPE on dental care may be attributed to variables not included in the survey. It may be beneficial to collect additional data in the framework of this national survey, such as general health status which has been shown to correlate with dental health and dental services utilization [4]. In addition, households only reported on dental expenses in the last 3 months, which may not reflect accurately their OOPE. Since there is high variability in the frequency of visits to the dentist, it may be that a period of 6 months would be a better timeframe for reporting.

## Conclusions

OOPE for dental care are still a burden for Israeli households. While some benefit from the ongoing reform between the two surveys was apparent, disparities were still observed according to different demographic and socioeconomic characteristics. Extending the dental health reform to include older segments of the population and addressing barriers to preventive dental care, especially among Arabs and those with a lower educational level, may help in lowering this economic burden and decrease inequalities related to dental health. The Israeli experience provides new insights into the determinants of OOPE for dental care. These are especially relevant for countries with a mixed public-private oral health system looking for strategies to reduce social inequalities in oral health.

## Data Availability

The data were obtained from the Household Expenditure Survey, conducted by the Israel Central Bureau of Statistics (ICBS) in 2014. Restrictions apply to the availability of these data, which were used under license for the current study, and so are not publicly available. Data are however available from the ICBS to any scientist wishing to use them, without breaching participant confidentiality.

## Abbreviations

CDBS: Child Dental Benefits Schedule
CHIP: Children’s Health Insurance Program
CI: Confidence Interval
DALYs: Disability-Adjusted Life-Years
ICBS: Israel Central Bureau of Statistics
NHI: National Health Insurance
NIS: New Israeli Shekels
OOPE: Out-of-Pocket Expenditure
OR: Odds Ratio
SES: Socioeconomic Status
USD: United States Dollars
